# Mitochondrial Graph-Based Pan-Genome Analysis of *Hypsizygus marmoreus*: Structural Variation, Adaptive Evolution, and Its Implications for Germplasm Resource Improvement

**DOI:** 10.3390/ijms27073129

**Published:** 2026-03-30

**Authors:** Ruichen Ma, Wenyun Li, Yongmei Miao, Ruiheng Yang, Youran Shao, Junjun Shang, Yan Li, Yuan Gao, Dapeng Bao, Yingying Wu

**Affiliations:** 1 College of Agriculture, Anhui Science and Technology University, Fengyang 233100, China; mx1272493931@outlook.com; 2 National Engineering Research Center of Edible Fungi, Key Laboratory of Applied Mycological Resources and Utilization, Ministry of Agriculture, Institute of Edible Fungi, Shanghai Academy of Agricultural Sciences, Shanghai 201403, China; liwenyun1209@126.com (W.L.);; 3 College of Biomedical and Health Science, Anhui Science and Technology University, Fengyang 233100, China; mym416@163.com; 4 Shanghai Key Laboratory of Agricultural Genetics and Breeding, Shanghai Academy of Agricultural Sciences, Shanghai 201106, China

**Keywords:** *Hypsizygus marmoreus*, mitochondria, structural variation, graph-based pan-genome, fungal genetics

## Abstract

Mitochondria regulate nuclear genomes and their own genetic material, primarily to provide energy in eukaryotes. Currently, high-throughput sequencing technologies are being used to resolve the mitochondrial genomes of various edible fungi. However, the application of pan-genomes for the analysis of edible mushroom mitochondrial genomes remains unexplored. In this study, we conducted a comparative mitochondrial genome analysis of 31 *Hypsizygus marmoreus* strains (four newly sequenced monotypes and 27 public datasets), ranging from 98,284 to 111,087 bp. This variation was determined to be primarily driven by dynamic changes in non-coding regions, particularly intronic polymorphisms in the *cox1* gene. Further, transfer RNA (tRNA) secondary structures exhibited atypical globular and elongated conformations alongside copy number variations. Additionally, codon usage showed a pronounced A/T bias, whereas core respiratory chain genes demonstrated an evolutionary pattern of strong purifying selection. Furthermore, the 31 mitochondrial genomes of *H. marmoreus* were found to harbor eight gene rearrangement patterns and five genetic clusters, and the pan-genome analysis (220,364 bp, 217 nodes) captured abundant single-nucleotide polymorphisms (SNPs), insertions/deletions (InDels), and structural variations. This study provides breeding-relevant genetic markers and a genomic framework for *H. marmoreus* germplasm classification, genetic improvements, and the molecular breeding of stress-resilient varieties.

## 1. Introduction

*Hypsizygus marmoreus*, an edible macrofungus belonging to the Basidiomycota phylum, Agaricomycetes class, Agaricales order, Lyophyllaceae family, and *Hypsizygus* genus, is commercially cultivated as white and brown strains [1]. Moreover, it is very popular in East and Southeast Asia. Owing to its smooth, crisp, and tender texture, unique aroma, and shape similar to that of *Tricholoma matsutake*, it is called “false *T. matsutake*” in Japan. Previous studies on *H. marmoreus* have primarily focused on functional genes [2], nutrients, bioactive compounds [3], and mating-type genes [4]. Although multiple mitochondrial genomes of *H. marmoreus* have been sequenced and published, previous studies have mostly concentrated on the annotation of individual genomes or have comprised limited comparative analyses [5,6]. In addition, a graph-based pan-genomic perspective to systematically elucidate the intraspecific structural variation in *H. marmoreus* is still lacking, and the application of mitochondrial genomic variation to its germplasm classification and molecular breeding has not been explored. Graph-structured pan-genomes can efficiently store and visualize complex sequence variations (single-nucleotide polymorphisms and indels) and SVs (gene rearrangements and insertions/deletions), thereby substantially enhancing the ability to analyze genomic structure dynamics [7]. A graph-based pan-genome integrates multiple genomes into a single graph structure, enabling a more comprehensive representation of genetic diversity than a single reference genome [8]. As a commercially important edible fungus, the innovation of *H. marmoreus* germplasm resources and the development of molecular breeding technology are essential to improve its cultivation efficiency and stress resistance, and the mitochondrial genome, as a key cytoplasmic genetic resource, is of great significance for its molecular regulation and germplasm preservation.

The mitochondrial genome, a segment of genetic material located within mitochondria, encodes key proteins and RNAs involved in cellular energy metabolism [9]. In fungi, this structure has a ring shape, interacting with and remaining isolated from the nuclear genome. Its main distinguishing characteristic is that its rate of sequence variation is relatively fast and considerably higher than that of the nuclear genome, whereas it is relatively conserved in terms of protein-encoding elements [10]. Kim et al. analyzed tandem repeat sequences and *A*-matching types of mitochondrial genes in *Lentinus edodes*, hypothesized that the differences in tandem repeats were closely related to the evolutionary process, and used these tandem repeat sequences for molecular labeling to screen for suitable high-potential strains with nuclear-cytoplasmic matching [11]. Song et al. replaced the mitochondria within *L. edodes* cells and analyzed the effect of browning during the coloring stage based on transcriptome data [12]. They found that the mitochondria were associated with the color and stem length of *L. edodes*. Wu et al. conducted a comparative analysis of intron dynamics and gene rearrangements in the mitochondrial genomes of two saprotrophic *Coniophora* species, which could aid in understanding the origin and evolutionary patterns of boletes [13]. Tan et al. conducted a comparative analysis of mitochondrial genomes from 46 strains of *Flammulina filiformis*, identified polymorphisms in the *cox1* intron and intergenic regions, and designed primers to identify mitochondrial differentiation [14]. Moreover, the mitochondrial genomes of 361 *Agaricus bisporus* individuals have revealed the global evolutionary process and domestication history of this species [15]. These studies show that the mitochondrial genome not only contains important genes related to the growth and development of edible fungi but also serves as a valuable resource for studying population genetics and evolutionary history.

With the decline in sequencing costs and innovation of analytical methods, pan-genome research has expanded from nuclear genomes to organelle genomes. The pan-mitogenome can systematically reveal the genetic diversity, adaptability, and evolutionary mechanisms of a species by integrating the mitochondrial information of multiple strains [16,17]. Although this method has shown potential in medical research related to animals, plants, and humans, it is still underdeveloped in the field of edible fungi, especially in research related to mitochondrial SVs in *H. marmoreus*.

To investigate the pan-mitogenome characteristics of *H. marmoreus*, we performed whole-genome sequencing of two pairs of compatible monokaryons (f2, f4, nn12-1, and nn12-17) derived from two commercial strains with superior traits. These data were integrated with 27 publicly available *H. marmoreus* genomic datasets in the National Center for Biotechnology Information (NCBI) database. A comprehensive analysis of the compositions of mitochondrial protein-coding genes (PCGs), tRNA secondary structures, repetitive sequences, codon usage bias, evolutionary selection pressure, and nucleotide diversity was further conducted. Subsequently, we employed phylogenetic reconstruction, population structure analysis, and mitochondrial graph-based pan-genome methods to systematically explore the evolutionary patterns and genetic variation profiles of the *H. marmoreus* mitogenome. Ultimately, the aim of this study was to construct the first graph-based mitochondrial pan-genome of *H. marmoreus*, explore its evolutionary characteristics and structural variation rules, and provide breeding-relevant genetic markers and a genomic framework for *H. marmoreus* germplasm improvements, molecular breeding, and germplasm preservation.

## 2. Results

### 2.1. Sequence Characteristics of the Mitochondrial Pan-Genome of H. marmoreus

**Figure 1:**
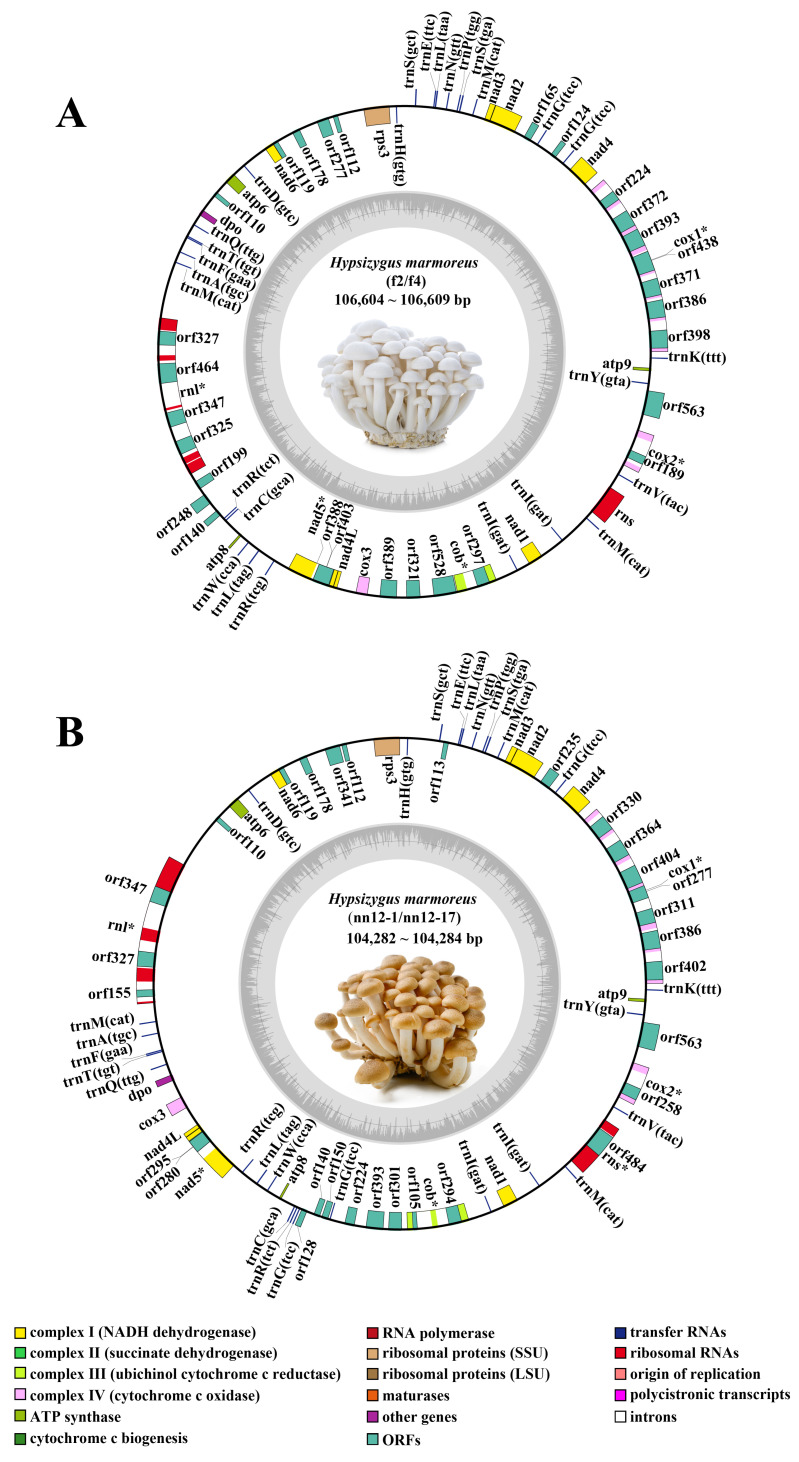
Mitochondrial genome map of the *Hypsizygus marmoreus*. The map in (**A**) represents white *H. marmoreus*, whereas that in (**B**) represents brown *H. marmoreus*. Genes displayed outside the circle are transcribed in a clockwise direction, whereas those inside the circle are transcribed in a counterclockwise direction. Different colors indicate different functional genes. The GC content is represented by a dark gray graph on the inner circle. Asterisks (*) indicate intron-containing genes.

Based on sequence homology with the *H. marmoreus* mitochondrial genome (GenBank: MF133443.1) [6], mitochondrial genomes of 106,605 bp (f2) and 106,609 bp (f4) were identified in the white varieties, whereas those of 104,282 bp (nn12-1) and 104,284 bp (nn12-17) were identified in the brown varieties (Figure 1). All four complete mitochondrial genomes from *H. marmoreus* strains were circular DNA molecules with GC contents ranging from 31.52% to 31.72%, containing 41 to 49 annotated genes. Compared to those of other edible fungi, the mitochondrial genome size of *H. marmoreus* was comparable to those of *Sanghuangporus sanghuang* (112,060 bp) and *Antrodia cinnamomea* (115,207 bp) but larger than those of *Schizophyllum commune* (49,704 bp), *Pleurotus ostreatus* (65,311 bp), *Volvariella volvacea* (65,668 bp), *Pleurotus eryngii* (72,650 bp), *Hericium erinaceus* (83,518 bp), and *F. filiformis* (88,513 bp) and smaller than those of *L. edodes* (121,685 bp), *A. bisporus* (135,005 bp), and *Grifola frondosa* (197,486 bp) [18]. The significant differences in mitochondrial genome sizes among different edible fungi may be attributed to diversity in the genome structure, including variations in the number and arrangement of core genes, as well as the number and types of introns [19].

Simultaneously, based on the assembly of 27 public datasets of *H. marmoreus* downloaded from NCBI and combined with the mitochondrial genomes assembled from the four strains for a joint analysis, the size range of the 31 mitochondrial genomes was found to be 98,284 bp to 111,087 bp, with the GC content ranging from 31.52% to 32.12%. Moreover, they contained 39–49 annotated genes (Table 1). In total, 15 conserved PCGs were identified in the mitochondrial genomes of the 31 strains. These genes have important functions in the electron transport chain and oxidative respiration, including seven NADH dehydrogenase-coding genes (*nad1*, *nad2*, *nad3*, *nad4*, *nad4L*, *nad5*, and *nad6*). The mitochondrial genomes also contained the following genes: a gene encoding a subunit of complex III (ubiquinol–cytochrome c reductase), one cytochrome b-encoding gene (*cob*); three cytochrome c oxidase genes (*cox1*, *cox2*, and *cox3*) involved in the respiratory electron transport chain; three ATP synthase subunit-coding genes (*atp6*, *atp8*, and *atp9*); and one ribosomal protein S3-coding gene (*rps3*). In addition, during re-annotation of the 31 *H. marmoreus* mitochondrial genomes, a DNA polymerase-encoding gene (*dpo*) was identified in 27 strains, whereas it was not detected in the remaining four strains, indicating the existence of a strain-specific deletion. These results indicate that there are significant intraspecific variations in the sizes and numbers of genes in the mitochondrial genome of *H. marmoreus*, providing a basis for a subsequent exploration of its structural dynamics and evolutionary mechanisms. These intraspecific variations in mitochondrial genome size and gene number provide an important genetic basis for *H. marmoreus* germplasm classification and genetic diversity evaluations.

### 2.2. Secondary Structure Analysis of tRNAs in the Mitochondrial Genome of H. marmoreus

**Figure 2:**
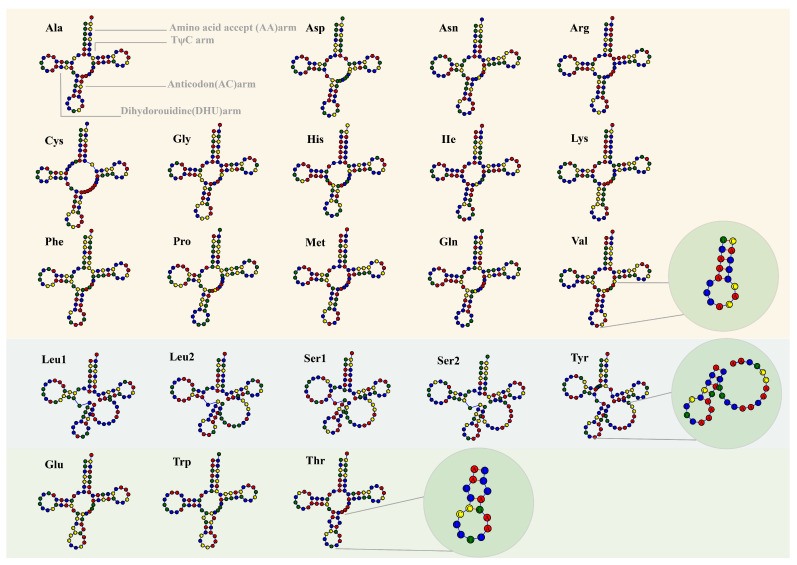
tRNA secondary structure prediction map of the 20 amino acids encoded by the mitochondrial genome of *Hypsizygus marmoreus*. The red, blue, green, and yellow spheres represent the bases A, U, G, and C, respectively.

tRNAs are key for deciphering the genetic code of mRNA and mediating protein synthesis. The typical cloverleaf-shaped structure conforms to the conserved characteristics of mitochondrial tRNA, and the additional spherical structure expands the conformational spectrum of mitochondrial tRNA. In this study, the mitochondrial genomes of the 31 strains were found to contain 25–28 tRNAs, mainly composed of 22 core tRNAs and some of their replicas, collectively responsible for the transport of 20 amino acids (Figure 2). The secondary structure prediction results showed that 14 tRNA molecules had typical cloverleaf structures, whereas five tRNAs (tRNA-Leu1, tRNA-Leu2, tRNA-Ser1, tRNA-Ser2, and tRNA-Tyr) had expanded ring structures at the DHU or TΨC ring. Three tRNAs (tRNA-Thr, tRNA-Trp, and tRNA-Glu) had atypical, elongated arms or long-strip conformations. Two types of structural variation were observed in this region: an expanded conformation at the junction between the anticodon arm and the TΨC arm and a minor local change in the anticodon arm. A copy number analysis indicated that all strains had three copies of tRNA-Met and that most strains had two copies of tRNA-Gly, but the nn12-1 and nn12-17 strains had three copies. These results revealed significant diversity in the secondary structure and copy numbers of tRNAs in the mitochondria of *H. marmoreus*, suggesting that it may be related to adaptive evolution or functional differentiation processes.

### 2.3. Analysis of Repeat Sequence Characteristics in H. marmoreus Mitochondria

**Figure 3:**
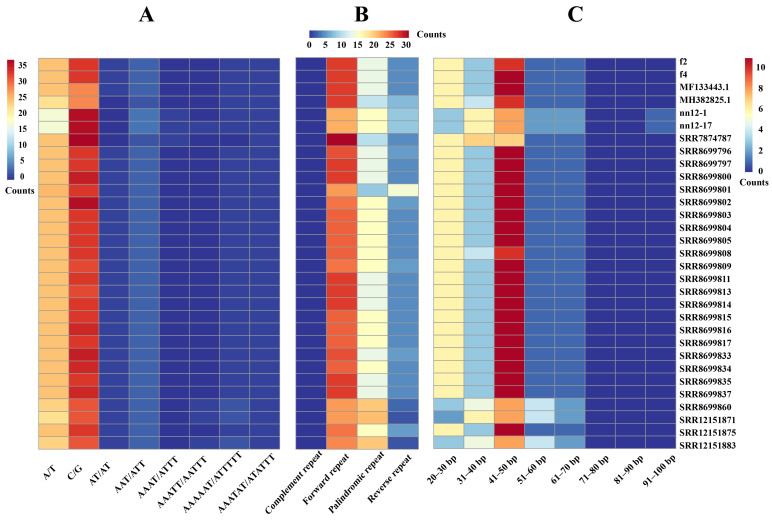
Heatmap of annotated repetitive sequences in the mitochondrial genome of *Hypsizygus marmoreus*. (**A**) Eight simple sequence repeat (SSR) motifs detected; (**B**) interspersed repeat; (**C**) long tandem repeat sequences, ranging from 20 bp to 100 bp, with increments of 10 bp.

Investigating the characteristics of mitochondrial repetitive sequences in *H. marmoreus* has helped to understand the evolutionary selection preferences for different repeat types, elucidate the associated genetic mechanisms, and facilitate germplasm resource identification. In this study, simple sequence repeat (SSR) detection, REPuter (available online: ‘https://bibiserv.cebitec.uni-bielefeld.de/reputer’; accessed on 5 October 2025) and TRF software, v 4.09 were employed to conduct a systematic analysis of various repetitive sequence types within the mitochondria of 31 *H. marmoreus* strains. SSR detection successfully revealed eight different SSR motifs (Figure 3A), including two mononucleotide SSRs, one dinucleotide SSR, one trinucleotide SSR, one tetranucleotide SSR, one pentanucleotide SSR, and two hexanucleotide SSRs. Among them, C/G motif variants were the most abundant, and the quantity varied significantly among strains (SRR7874787 contained 38, while MF133443.1 and MH382825.1 had only 29). The abundance of A/T-type motifs was the second highest (average 24.4). Notably, tetranucleotide motifs (AAAT/ATTT) were present in only three samples, and pentanucleotide motifs (AAATT/AATTT) were found in only four samples, all as single copies. The enrichment of C/G motifs appears to be a common feature of the mitogenomes of edible fungi. For example, in *L. edodes*, C/G-type SSRs account for more than 60% of total SSRs, and variations in their tandem repeats have been linked to evolutionary pathways and nuclear–cytoplasmic compatibility [20]. Similarly, a large-scale pan-mitogenome study of 361 *A. bisporus* strains demonstrated that a high frequency of C/G motifs correlates positively with genomic stability, potentially because they form stronger hydrogen bonds, thereby contributing to DNA duplex stability [15].

An analysis using REPuter software revealed significant differences in the abundances of four types of repetitive sequences, specifically forward (F), palindromic (P), reverse (R), and complementary (C), within the mitochondrial genome of *H. marmoreus*. These sequences exhibited the following distribution pattern: F > P > R > C (Figure 3B). At the individual sample level, the abundance of each repeat type varied markedly among strains. Notably, sample SRR8699801 contained only nine palindromic repeats (P), which was significantly lower than the average across all samples, whereas its reverse repeats (R) reached 16 occurrences, which was markedly higher than the reverse repeat frequency observed in all other samples.

Long tandem repeat sequences ranging from 20 to 100 bp were identified using TRF software. The results showed that repeat sequences of different length ranges presented different distribution patterns in the mitochondria of *H. marmoreus* (Figure 3C). Among all samples, the content of repeat sequences with a length of 41–50 bp was the highest, with an average of 10.2 repeats, and this length reached 11 repeats in 21 samples. The abundance of repeat sequences with a length of 20–30 bp ranked second, with an average of 5.5 repeats. Moreover, among the 26 samples, the number of repeats was relatively high, reaching six repeats. Notably, in the 71–90 bp-length range, no repeat sequences were found in any of the samples. In the 91–100 bp-length range, only two strains, nn12-1 and nn12-17, each contained one repeat. No repeat sequences within this range were detected in the remaining samples. In conclusion, the mitochondrial repeat sequences of *H. marmoreus* showed a high degree of polymorphism in their type, abundance, and distribution. The dynamic changes in these repeat elements may be one of the important forces driving genome size variation and structural evolution. The high polymorphism of mitochondrial repetitive sequences in *H. marmoreus* can be used as molecular markers for germplasm identification and genetic diversity analysis of this species.

### 2.4. Analysis of Codon Usage Preferences in the Mitochondrial Genome of H. marmoreus

**Figure 4:**
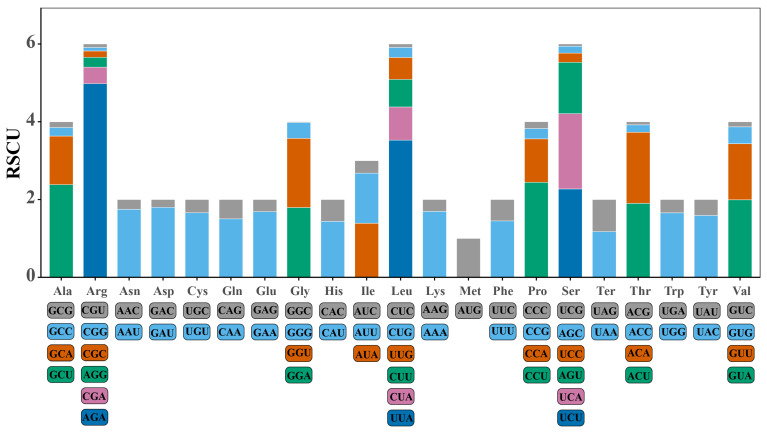
Relative synonymous codon usage (RSCU) analysis of the mitochondrial genome of *Hypsizygus marmoreus*.

The codons in the mitochondrial genome of *H. marmoreus* were analyzed based on the relative synonymous codon usage (RSCU), and a significant bias was found in the use of synonymous codons for most amino acids (Figure 4). From the perspective of amino acid codon preference, UUU (RSCU = 1.45) for phenylalanine and AUU (RSCU = 1.29) for isoleucine were used more frequently, reflecting a preference for U at the third codon position. AGA (RSCU = 4.98) for arginine, UUA (RSCU = 3.53) for leucine, and AAA (RSCU = 1.70) for lysine were also used more frequently, indicating a preference for A at the third codon position. Furthermore, the dominant codons of most amino acids, such as proline, valine, and threonine, reflected a strong A/T base preference in the mitochondrial genome, which is consistent with previous findings that the mitochondrial PCG synonymous codons in eight *Pleurotus* strains also exhibited a preference for ending with A/T [21].

### 2.5. Analysis of Substitution Rates in the Mitochondrial PCGs of H. marmoreus

**Figure 5:**
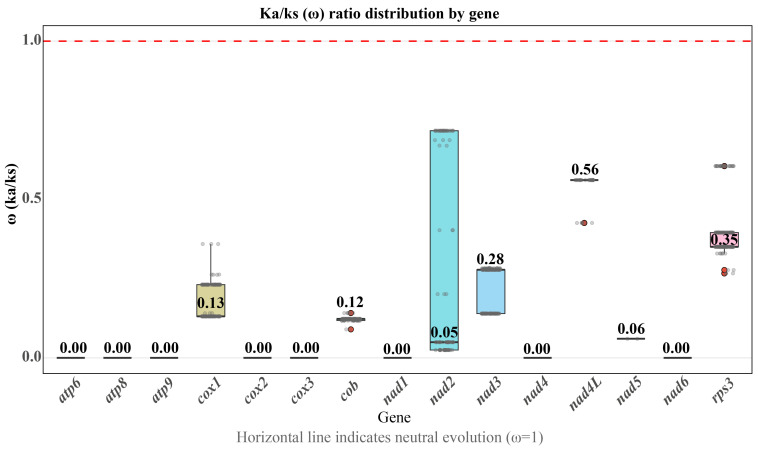
Selective pressure analysis of 15 core protein-encoding genes in the mitochondrial genomes of 31 *Hypsizygus marmoreus* strains.

The non-synonymous and synonymous substitution (Ka/Ks) ratio was used to show the differences in evolutionary constraints among 15 conserved PCGs of 31 *H. marmoreus* strains (Figure 5). The ω values of *cox1*, *cob*, *nad2*, *nad3*, *nad4L*, *nad5*, and *rps3* ranged between 0 and 1, whereas the remaining eight genes exhibited values close to 0 (ω ≈ 0), indicating that purifying selection is the dominant force during the evolution of these core genes. The *cox1* (ω = 0.131) and *cob* (ω = 0.123) genes, which encode cytochrome c oxidase subunit I and cytochrome b, respectively, showed strong evolutionary conservation. This is consistent with the essential requirement for a stable and efficient respiratory chain to support mycelial growth and fruiting body development in *H. marmoreus*, a wood-decaying fungus that relies on lignocellulose decomposition for energy. The genes *nad2* (ω = 0.049), *nad3* (ω = 0.278), *nad4L* (ω = 0.561), and *nad5* (ω = 0.06), which encode subunits of the mitochondrial NADH dehydrogenase complex, also exhibited low Ka/Ks ratios. This complex is crucial for electron transfer in the respiratory chain, and even minor amino acid changes disrupt its structure and impair energy production, suggesting strong purifying selection. Furthermore, *atp8* and *cox3* were completely conserved across all 31 strains, whereas *atp6*, *atp9*, *nad1*, *nad4*, and *nad6* only contained synonymous substitutions. Collectively, these patterns indicated intense purifying selection and high functional constraints. This finding is consistent with findings in other basidiomycetes, where the overall Ka/Ks values of 14 core PCGs in five *Ganoderma* species [22] and five *Boletus* species [23] were also less than 1, confirming that purifying selection is the dominant evolutionary force acting on essential mitochondrial respiratory genes.

### 2.6. Analysis of Nucleic Acid Diversity in the Mitochondria of H. marmoreus

**Figure 6:**
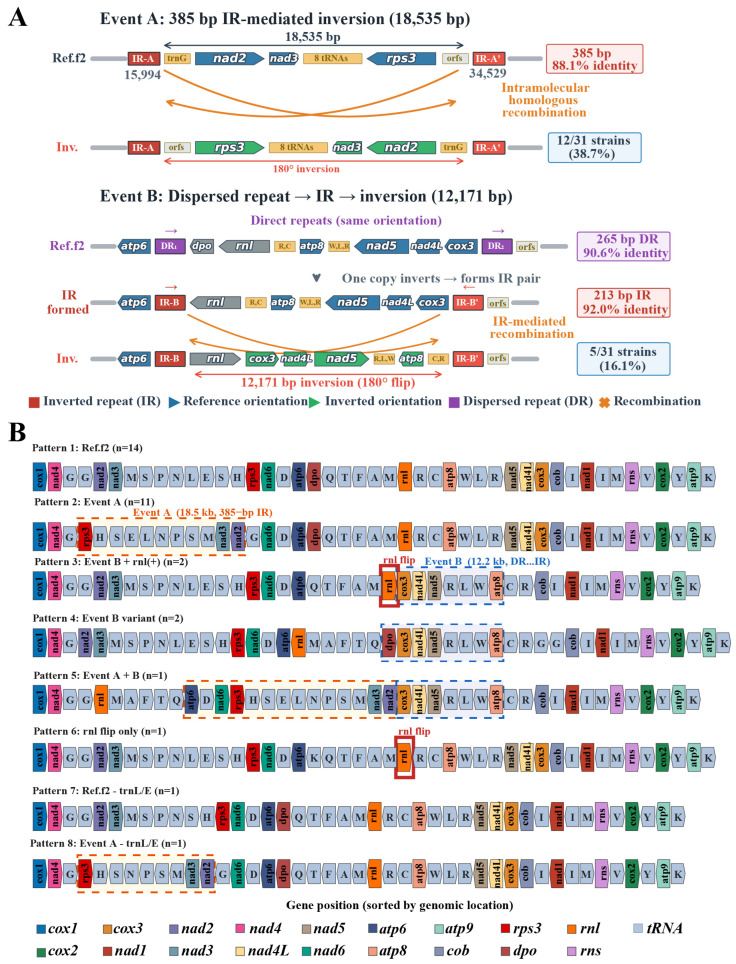
Mitochondrial genome rearrangement events and gene order patterns in *Hypsizygus marmoreus*. (**A**) Two major molecular events driving large-scale structural rearrangements in the mitochondrial genome of *H. marmoreus*; (**B**) eight unique gene arrangement patterns identified in 31 strains of *H. marmoreus*. The different colors in the figure represent different genetic types. Among them, the light blue blocks represent the single-letter abbreviations encoded by various tRNAs. The colored arrows indicate transcription direction. The red dashed boxes denote Event A, the blue dashed boxes denote Event B, and the red solid boxes indicate changes in the transcription direction of rnl.

An analysis of the core genes revealed low nucleotide diversity (Pi < 0.03) across most of them (Figure S1), with only the *nad2* gene having a relatively higher value. This indicates that the core functions of the respiratory chain are subject to intense purifying selection, whereas genes and non-coding sequences exhibit distinct evolutionary rates. In the mitochondrial genomes of 31 *H. marmoreus* strains, we observed large-scale structural rearrangements driven by two major molecular events (Figure 6A). Event A is an 18,535 bp inverted repeat (IR)-mediated inversion involving the *nad2–nad3–rps3* region, mediated by a pair of 385 bp IRs with 88.1% identity, occurring in 38.7% of the strains. Event B is a 12,171 bp inversion of the *atp8*–*nad5*–*nad4L*–*cox3* region, occurring in 16.1% of strains. In the reference genome (f2), a pair of 265 bp direct repeats (90.6% identity) was found to flank the *dpo* and *cox3* regions. In inverted strains, these dispersed repeats have evolved into a 213 bp inverted repeat pair (IR, 92.0% identity), which subsequently mediated the segmental inversion through intramolecular homologous recombination. Based on gene arrangement patterns, we classified the 31 strains into eight distinct gene orders (Figure 6B), including six core rearrangement patterns and two cases caused by tRNA loss. Pattern 1 represents the reference type (Ref.f2), containing 14 strains; Pattern 2 corresponds to the Event A inversion (11 strains); Pattern 3 is a combination of Event B and an *rnl* inversion (two strains); Pattern 4 is a variant of Event B (2 strains); Pattern 5 is a combined inversion of Event A and B (one strain); Pattern 6 is an *rnl* inversion alone (one strain); and Patterns 7 and 8 represent *trnL/E* loss combined with Event A or the reference type, respectively (one strain each). These patterns suggest that mitochondrial genome rearrangements may enhance adaptability by altering gene expression patterns or through the generation of new gene combinations. The identified genetic variation hotspots and conserved regions provide candidate sites for the development of molecular markers for *H. marmoreus* molecular breeding.

### 2.7. Phylogenetic and Population Structure Analysis of H. marmoreus

**Figure 7:**
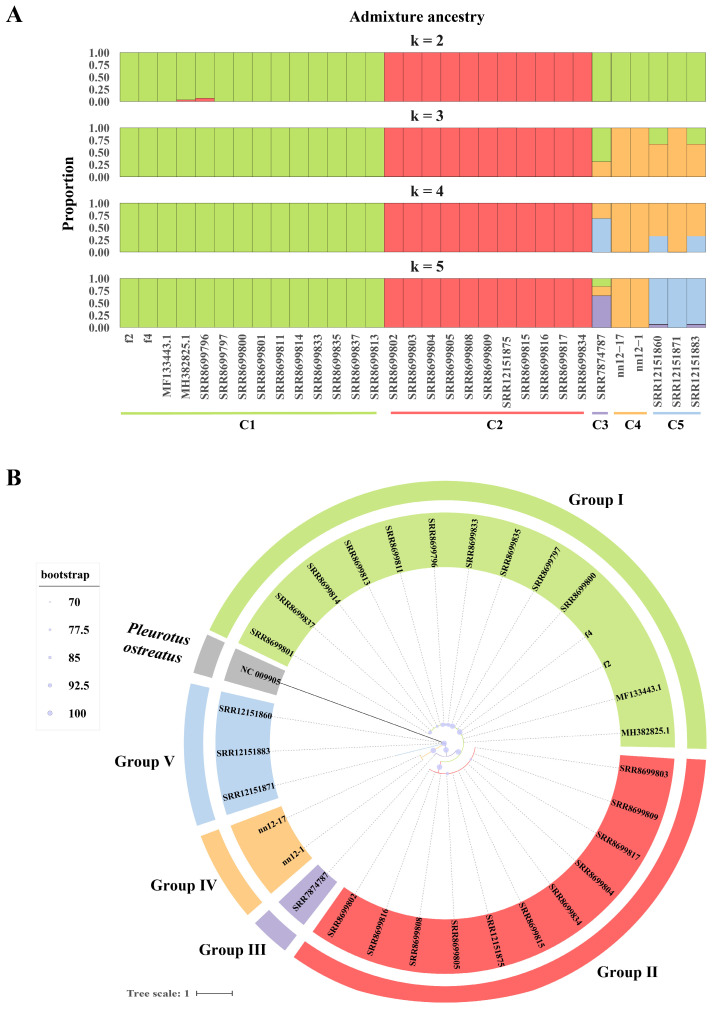
Phylogenetic and population structure analysis of *Hypsizygus marmoreus*. (**A**) Population structure bar plots at K = 2–5; (**B**) phylogenetic tree based on mitochondrial genomic SNPs.

Based on single-nucleotide polymorphism (SNP) data from 31 mitochondrial genomes of *H. marmoreus*, we determined its population structure characteristics. An ADMIXTURE analysis showed that when K = 5, the cross-validation error was the lowest (Figure S2), indicating that K = 5 was the optimal clustering level for the SNP matrix. This result suggested that the samples could be divided into five major genetic clusters (Figure 7A). Among them, the genetic backgrounds of clusters C1, C2, and C4 were relatively pure, whereas some individuals in clusters C3 and C5, such as the SRR12151860 strain, showed mixed genetic components derived from multiple ancestral groups. A phylogenetic tree analysis, using *P. ostreatus* (GenBank: NC009905) as an outgroup, further supported this classification, with a relatively high bootstrap support rate (>95%) among the different branches, suggesting that these lineages have undergone relatively independent genetic drift and fixation events during their evolutionary history (Figure 7B). The five genetic clusters and obvious population differentiation provide a theoretical basis for *H. marmoreus* selective breeding and germplasm innovation.

### 2.8. Analysis of the Pan-Genome of H. marmoreus Mitochondrial DNA

**Figure 8:**
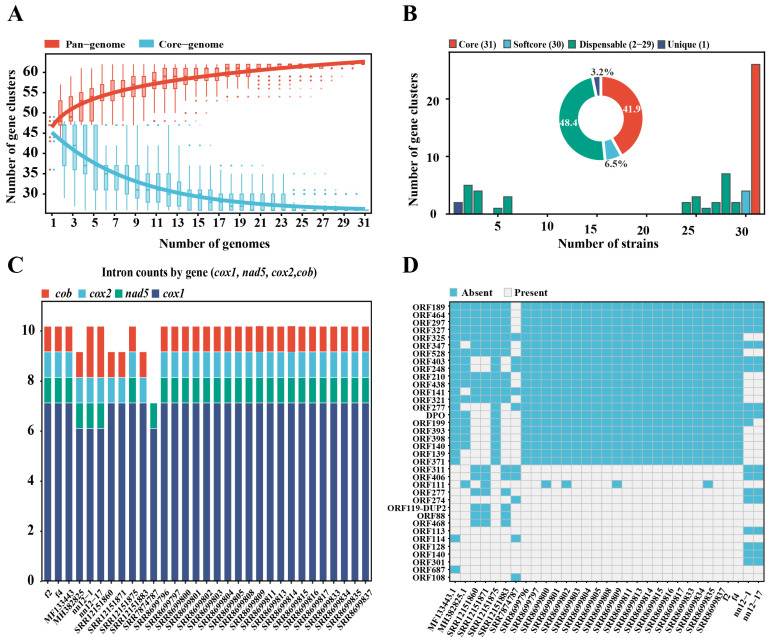
Analysis of mitochondrial pan-genome and structural variation characteristics of *Hypsizygus marmoreus*. (**A**) Profiling of the mitochondrial pan-gene pool; (**B**) distribution of gene families and compositions of core, soft-core, accessory, and strain-specific genes across the 31 mitogenomes; (**C**) distribution of intron numbers in the *cox1*, *nad5*, *cox2*, and *cob* genes across the 31 strains; (**D**) presence–absence variation profile of genes in the 31 *H. marmoreus* mitogenomes. The blue square indicates the presence of the corresponding homologous gene in the specific strain.

The pan-genome population growth curve showed that when the number of strains increased to approximately 25–28, the pan-genome entered a plateau phase (power-law fit: y = 46.48 × x^0.087^ (B < 1)), indicating that the pan-genome of this species tends to be near-closed (Figure 8A). Based on the frequency of gene families across the 31 genomes, 62 total gene clusters were identified and classified into four categories: core genes (26, accounting for 41.9%), soft-core genes (4, 6.5%), dispensable genes (30, 48.4%), and unique genes (2, 3.2%) (Figure 8B) [24]. In previous studies, differences in the mitochondrial genome size were mainly associated with intron insertion polymorphisms, repetitive sequences, and large-fragment SVs. In *H. marmoreus*, the mitochondrial genome sizes were mainly distributed in three intervals: 98 kb, 102–106 kb, and 111 kb (Figure S3). A further analysis revealed that among the 31 strains, there were significant differences in the number of introns in four genes, namely *cox1*, *nad5*, *cox2*, and *cob*. Among these, the *cox1* gene contained the most introns, with most strains harboring seven introns. However, the *cob* and *cox2* genes of SRR7874787 contained no introns. The genome of this strain was the shortest among all strains, which is consistent with a significant association between intron deletions and genome length (Figure 8C).

An analysis based on orthologous groups revealed that non-core variable genes exhibited pronounced presence–absence variation among different strains (Figure 8D), indicating heterogeneous conservation patterns across the mitochondrial pan-genome. Specifically, orf563, orf297/294, and orf321 were highly conserved and remained stable in most strains (28–29 of 31), whereas orf113, orf128, orf114, and orf301 were detected only sporadically in 2–3 strains, reflecting their strain-specific distribution. Notably, the *dpo* gene, encoding a DNA polymerase derived from mitochondrial plasmid integration, was absent in the four strains that had undergone IR-mediated genome rearrangements (SRR12151860, SRR12151871, SRR12151883, and SRR7874787), suggesting a potential association between large-scale structural variation and the loss of plasmid-derived sequences. In addition, several ORFs (including orf128, orf140, and orf301) were exclusively present in the nn12-1 and nn12-17 strains and localized to intergenic regions, implying rapid evolutionary dynamics that may contribute to mitochondrial genome diversification and functional differentiation.

**Figure 9:**
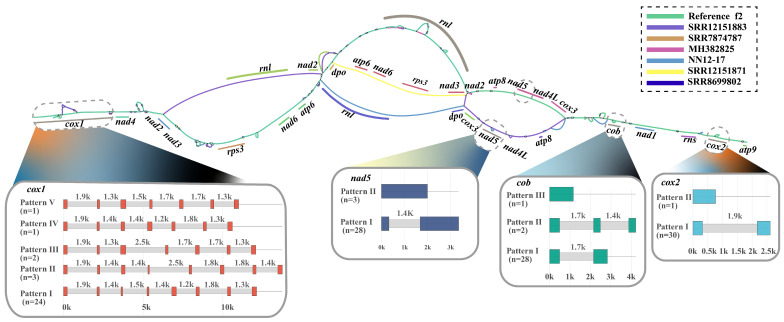
Analysis of the pan-genome and structural variation characteristics of the mitochondrial genome of *Hypsizygus marmoreus*. Construction of the mitochondrial pattern pan-genome of *H. marmoreus* based on Bandage software.

Based on 31 mitochondrial genomes of *H. marmoreus*, we successfully constructed a mitochondrial pattern pan-genome using the f2 strain as the reference. This pan-genome was generated in graphic fragment assembly (GFA) format, with a total length of 220,364 bp, containing 217 nodes and 293 edges. The overall structure reflected the SV characteristics of the mitochondrial genome. A BLAST localization analysis of core coding genes revealed that most core gene regions had not undergone significant SV, whereas genes containing introns exhibited large-fragment insertions/deletions (Figure 9). Specifically, the *cox1* gene region exhibited multiple bubble-like structures, mainly due to intron insertion and deletion events. Among the 31 strains of *H. marmoreus*, 27 (87.1%) maintained the canonical “7 introns + 8 exons” structure of the *cox1* gene, including 24 sharing identical intron insertion sites with the reference strain f2 and three rearranged strains (SRR12151860, SRR12151871, and SRR12151883) in which the seven introns were retained but positioned at partially different locations. The *nad5* gene was simultaneously affected by both gene rearrangement and intron variation. A sequence analysis revealed that 26 strains (83.9%) maintained the canonical antisense *nad5* arrangement carrying a 1363 bp intron. Two rearranged strains (nn12-1 and nn12-17) retained the intron (1447 bp) despite sense-strand inversion, whereas three rearranged strains (SRR12151860, SRR12151871, and SRR12151883) exhibited both orientation disruption and complete intron loss. The *cob* gene contained a single intron in 28 strains (90.3%), whereas nn12-1 and nn12-17 acquired an additional intron at the position 820 site, and SRR7874787 lacked introns entirely. The *cox2* gene carried a single intron in 30 strains (96.8%), with substantial length variation among strains. Aside from the genes described, no large-scale structural variations were detected in other mitochondrial coding regions.

In addition, 2323 variant sites were identified, including 1563 SNPs, 522 insertions and deletions (InDels), 246 polynucleotide polymorphisms, and 175 other complex variations (Figure S4A). The conversion to transmutation ratio was 820:690 (1.19:1), which was relatively low but conformed to the common conversion bias characteristics of the mitochondrial genome (Figure S4B), reflecting the high spontaneous mutation tendency of mitochondrial DNA (Figure S4C). The InDel analysis results showed that short fragment InDel events (≤5 bp) dominated. In total, 186 single-base deletions (−1) and 144 single-base insertions (+1) were detected. Variations were mainly distributed in the intergenic regions, followed by the coding regions. However, the variation density (number of variations per kb) in the rRNA region was slightly higher than that in the CDS region (Figure S4D). Specifically, C>T (239 times) and G>A (197 times) were the main mutation types, followed by A>G (187 times) and T>C (197 times) (Figure S4E), which was consistent with the typical mutation pattern caused by cytosine deamination rather than mutations resulting from external DNA damage or artificial mutagenesis [25]. In addition, multiple large-fragment deletions were identified. For example, 18 sites with 60 bp deletions were detected, suggesting the presence of structurally unstable regions in the mitochondrial pan-genome. The constructed graph-based mitochondrial pan-genome and identified abundant genetic variations provide a comprehensive genomic resource for molecular regulation and stress-resilient variety breeding for *H. marmoreus*.

## 3. Discussion

In this study, mitochondrial genomes from four mononuclear strains of *H. marmoreus* were sequenced and assembled, and, together with publicly available NCBI datasets, 31 total mitochondrial genomes were integrated to construct a mitochondrial pan-genome map of *H. marmoreus*. Unlike previous mitochondrial studies on edible fungi, such as *F. filiformis* and *A. bisporus*, which were based on the assembly of a single strain or multiple strains, the graphical mitochondrial pan-genome generated here provides a more systematic and comprehensive analysis of mitochondrial structural diversity, intron dynamics, population structures, and evolutionary trends. Throughout evolution, the mitochondrial genomes of animals, fungi, and plants have diverged significantly. Animal mitochondrial genomes exhibit highly conserved structures with stable gene orders but elevated point mutation rates [26], whereas plant mitochondrial genomes display extremely low point mutation rates but high structural variability [27]. The evolutionary characteristics of fungal mitochondria lie between these two extremes, with relatively conserved coding genes but substantial variation in the genome structure and size [28]. Previous studies have shown that fungal mitochondrial genomes are among the most variable groups of eukaryotes, primarily owing to factors such as dynamic intron changes, the accumulation of repetitive sequences, and the presence of non-conservative PCGs [29,30,31]. In this study, variations in the mitochondrial genome size of *H. marmoreus* (98,284–111,087 bp) were found to primarily originate from intron acquisition and loss, variations in repetitive sequences, and the insertion or deletion of *dpo* genes, which provide the structural basis for subsequent rearrangement patterns. Studies have shown that *dpo* sequences derived from linear plasmids are often integrated into mtDNA, with insertion sites frequently located in genomic rearrangement hotspots. This is consistent with our observations based on the *nad2*–*rps3* (Event A) and *atp8*–*cox3* (Event B) regions. These integrations are closely associated with genomic expansion and repetitive sequence enrichment; crucially, our results confirm that the enriched IRs function as the primary drivers of large-scale structural rearrangements via intramolecular homologous recombination [32]. Furthermore, studies on *Agrocybe aegerita* have shown that the *polB* gene may originate from linear mitochondria and, after integration, promote SVs such as local duplication or inversion [33]. These results are consistent with the gene presence/absence polymorphisms and rearrangement phenomena observed in the mitochondria of Basidiomycetes, suggesting that IR-mediated recombination, potentially triggered by the insertion of plasmid-derived mobile elements, is a key mechanism driving structural heterogeneity and rearrangements in basidiomycete mitochondria.

Most eukaryotic ancestors obtained their mitochondrial genomes from Alphaproteobacteria through an endosymbiotic mechanism. During subsequent evolution, most mitochondrial genes gradually integrated into the nuclear genome, with only a set of core and non-conserved PCGs retained to maintain the stability of the oxidative phosphorylation process [34]. Among the 31 *H. marmoreus* strains studied, all mitochondrial genomes contained 15 PCGs. These genes exhibited a distinct preference for A/T-terminating codons in amino acid usage, consistent with the codon preference patterns observed in the genus *Pleurotus* [21] and in wild *Auricularia villosula* [35]. This provides a basis for optimizing the codons of exogenous functional genes for genetic engineering breeding, enhancing the expression efficiency of exogenous genes, and accelerating the *H. marmoreus* breeding process. The Ka/Ks analysis showed that these genes have all been subjected to strong purifying selection (ω < 1) during evolution, consistent with reports of some genera such as *Ganoderma* and *Boletus* [36], indicating the key role of these conserved genes in mitochondrial energy metabolism. Notably, the *nad4L* (ω = 0.56) and *rps3* (ω = 0.35) genes exhibit relatively high evolutionary rates, a phenomenon potentially linked to their relaxed functional constraints and interactions with mobile genetic elements. *nad4L* encodes a small subunit of respiratory complex I, which tends to accumulate neutral mutations more readily than core subunits, resulting in a relatively higher evolutionary rate. *rps3* encodes a structural component of the small subunit of mitochondrial ribosomes; however, studies have indicated that during *L. edodes* color transformation, it may participate in regulating non-ribosomal functions, such as oxidative stress responses, energy metabolism coordination, and extracellular matrix remodeling. This suggests that, while maintaining fundamental translational functions, *rps3* may also play a regulatory role in environmental adaptation [12]. These genes with higher evolutionary rates accumulate neutral mutations, making them important candidate genes for genetic improvements to agronomic traits in *H. marmoreus*, such as stress tolerance and yields.

In this study, we constructed a graphical pan-genome of the *H. marmoreus* mitochondria, revealing abundant genetic variation (including SNPs, InDels, and SVs). Compared to a linear reference genome, this approach demonstrated significant advantages in variant detection, clearly illustrating large-scale SVs and intron insertion/deletion events within the *H. marmoreus* mitochondria. Notably, the respiratory chain-related gene *cox1* exhibited significant intronic polymorphisms. Such genes are widely reported as “high-frequency carriers” of intron insertions in Lycoperdaceae, *L. edodes*, *P. ostreatus*, *Armillaria*, and *F. filiformis* [14]. The numbers and types of introns in these genes far exceed those in other PCGs, suggesting that the *cox1* region has a greater capacity for intron accommodation, maintenance, and loss during the evolution of fungal mitochondrial genomes [37]. Notably, most introns in the mitochondrial genome of *H. marmoreus* were identified as Group I self-splicing endonuclease genes (HEGs). Their primary function involves homing endonucleases that cleave at homologous sites where introns are deleted, facilitating “homing” through the host DNA repair system. This process promotes the diffusion and maintenance of introns within populations and is accompanied by a series of sequence remodeling events, including local duplication, intergenic region expansion, and the recombination of adjacent ORFs, thereby amplifying mitochondrial genomic structural diversity [38]. Previous studies have demonstrated that, in various basidiomycetes, Group I introns rich in HEGs are frequently associated with significant structural rearrangements and segmental insertions/deletions. This leads to marked differentiation among strains in terms of their mitochondrial genome size, configuration, and gene linkage patterns [39]. These HEG-driven intronic dynamics and structural variations indirectly regulate mitochondrial energy metabolism and stress-response capabilities, thereby influencing strain growth rates and stress tolerance. For the molecular regulation of *H. marmoreus* cultivation, the identified *cox1* intronic polymorphisms and repetitive sequence dynamics can be used as key molecular regulation sites to regulate the energy metabolism of mycelium and fruiting body development, thereby improving its cultivation efficiency. In terms of germplasm preservation, the five genetic clusters identified in this study can be used as core germplasm resources, and the conserved mitochondrial genomic regions can be used as molecular markers for germplasm authenticity identification.

In conclusion, this study, through a mitochondrial pan-genome analysis, systematically revealed the evolutionary characteristics and functional adaptability of the *H. marmoreus* mitochondrial genome, providing a theoretical basis for variety breeding, molecular marker development, and genetic resource conservation. Specifically, 31 mitochondrial genomes ranging from 98,284 to 111,087 bp were integrated to construct a 220,364 bp graph-based pan-genome with 217 nodes, revealing eight gene rearrangement patterns and five genetic clusters. Future research could focus on the following directions: (1) investigating the mechanism of nucleo-mitochondrial interactions, combined with transcriptome-based or functional experiments, to explore the regulatory role of mitochondrial variations in phenotypes such as fruiting body development, and to develop specific molecular markers based on core variation sites for the precision molecular breeding of *H. marmoreus*; (2) expanding the application of the graphical pan-genome method to other edible and medicinal fungi and enabling analyses of evolutionary networks and the breeding history; (3) designing mitochondrial–nuclear and cytoplasmic exchange experiments, inspired by Cytoplasmic Hybrid studies in plants, to evaluate the direct effect of mitochondrial variations on phenotypes and environmental adaptability.

## 4. Materials and Methods

### 4.1. Experimental Strains and Data Sources

The monokaryotic strains of *H. marmoreus* (f2, f4, nn12-1, and nn12-17) used in this study, whose GenBank accession numbers are PV946885, PX600725, PX600726, and PX600727, respectively, are preserved by the National Edible Fungi Germplasm Resource Bank (Shanghai), Ministry of Agriculture and Rural Affairs, P.R. China. Among them, f2 and f4 comprise a pair of compatible monokaryons isolated from the white commercial strain of *H. marmoreus* Finc-W-247 (CGMCC 13193), which produces pure white, translucent fruiting bodies with regular, non-cracking pilei, has a long shelf life, and has crisp, tender, smooth flesh. Similarly, nn12-1 and nn12-17 comprise a pair of compatible monokaryons derived from the brown commercial strain *H. marmoreus* NN-12 (introduced from Japan), characterized by marbled pilei, a thick and firm texture, a long shelf life, and a distinctive crab-like flavor. Additionally, two complete mitochondrial genomes and 25 whole-genome sequencing datasets of *H. marmoreus* were downloaded from the NCBI database for subsequent integrated analysis [6].

### 4.2. Mycelium Collection

Strains f2, f4, nn12-1, and nn12-17 were inoculated onto PDA medium (39 g of potato dextrose agar powder, distilled water to 1 L, sterilized at 121 °C for 20 min before use). The cultures were incubated at 23 °C until mycelium growth reached the outer two-thirds of the 90 mm plate. Under sterile conditions, 10 g of the mycelium was scraped, rapidly frozen in liquid nitrogen, and stored at −80 °C for subsequent genomic sequencing.

### 4.3. Genome Sequencing, Assembly, and Annotation

Genome sequencing of the four experimental strains was performed using PacBio Sequel and Illumina NovaSeq platforms. The second-generation sequencing depth ranged from 30 to 41× with coverage of 1.23–1.65 GB, whereas the third-generation high-fidelity (HiFi) sequencing depth reached 100 to 175× with coverage of 4.0–6.9 GB. Based on this, the sequencing data of the mitochondrial genome obtained in this study have a coverage depth ranging from 2441 to 4616×, corresponding to an original data volume of 260.2–481.4 MB. Using the HiFi data obtained from sequencing, Flye v2.9.5-b1801 was employed for de novo whole-genome assembly [40]. The assembly results were visualized using Bandage v0.9.0 software [41]. Mitochondrial genome sequences were then extracted from the whole-genome data based on the sequencing depth, sequence length, and BLAST v 2.16.0+. Genome-wide resequencing data from 25 second-generation *H. marmoreus* samples were downloaded from NCBI and assembled using GetOrganelle v1.7.7.1 [42]. The mitochondrial genomes were annotated using MFannot [43] and Mitos [44]. For codon translation, the mitochondrial genetic code of molds/protozoa/coelenterates (transl_table = 4) was applied in both MFannot and Mitos. The annotation results were manually corrected using Geneious v2025.0.2 software to ensure their accuracy. The annotation results were visualized using OGDRAW v1.3.1. The secondary structure was predicted using tRNAscan-SE v2.0.12 [45], and a preliminary structure prediction graph was generated using RNAplot v2.7.0 [46] and finally visualized using a Python v 3.12.9 script.

### 4.4. Repeat Sequence Analysis

The MISA (MicroSatellite) Perl script was used to identify SSR loci in the mitochondrial genome sequences of all strains. Default parameters were applied, with the minimum repeat occurrences for 1–6 nt units set as 10, 6, 5, 5, 5, and 5, respectively [47]. Forward (F), palindromic (P), reverse (R), and complementary (C) repeat sequences were detected using the REPuter online tool, with a minimum repeat size set to 30 bp and an edit distance of 0. Long tandem repeats (>6 bp) were detected using the TRF online tool, with all parameters set to default values (2, 7, 7, 10, 50, and 500). All repeat sequence statistics were visualized using R language v4.3.1.

### 4.5. Codon Preference Analysis

Based on the mitochondrial genome annotation results of *H. marmoreus*, 15 common PCG sequences were extracted using PhyloSuite v1.2.3 [48]. The RSCU value was calculated using MEGA v11 [49], where RSCU > 1 indicates that the codon was preferred for amino acids, and RSCU < 1 indicates that the codon was used less frequently than expected.

### 4.6. Ka/Ks Analysis

The conserved PCG sequences from the mitochondrial genomes of the 31 *H. marmoreus* strains were aligned using MAFFT v7.526 [50]. Subsequently, we calculated the non-synonymous substitution rate (Ka) and synonymous substitution rate (Ks) using pamlX v1.3.1 [51]. The Ka/Ks ratio was analyzed to assess evolutionary selection pressure.

### 4.7. SNP Analysis

SNP analyses were conducted at 15 PCG levels. Multiple sequence alignments were performed on the corresponding sequences using MAFFT v7.505 software [50]. SNPs were detected using DnaSP v6 [52], and the nucleotide polymorphism (π) and Watterson’s θ population genetic diversity parameters were calculated. Gene location information in the GB annotation file was read using a custom Python script, and, combined with SNP data, a mitochondrial genome rearrangement pattern diagram was drawn using ggplot2 v 4.0.1 (R package).

### 4.8. Population Structure Analysis

Single-nucleotide variant data were screened using SNP-sites v2.5.1 [53]. Population genetic structure analysis was conducted within a range of K values (2–8) using Admixture v1.3.0, and the optimal K value was determined through cross-validation [54]. The phylogenetic tree was constructed using the maximum likelihood method using IQ-TREE v2.0.7 software [55]. Based on mitochondrial SNP data from 31 *H. marmoreus* strains, with *P. ostreatus* (GenBank: NC009905) as the outgroup, the best-fit model was selected using ModelFinder, and branch support was estimated based on 1000 ultrafast bootstrap replicates. Visualization was performed using the iTOL online platform [56].

### 4.9. Mitochondrial Pan-Genome Analysis of H. marmoreus

Gene family clustering was performed on the protein sequences of the 31 strains using OrthoFinder v2.55 [57]. Based on the presence–absence patterns of gene families, simulations of the pan-genome and core genome were performed using a custom script to calculate the number and distribution of core genes, non-core genes, and private gene families across strains. To construct the high-resolution variation map of the mitochondrial pangenome of *H. marmoreus*, the mitochondrial sequence of strain f2 was used as a reference, and the Minigraph-Cactus pangenome pipeline (v2.9.3) was employed, starting with Minigraph to efficiently capture SVs and construct the initial topological framework [58]. Subsequently, progressive multi-sequence alignment was performed using Cactus v7.0.0 software to generate a comprehensive graph pan-genome file (GFA format) and the corresponding hierarchical alignment file. On this basis, the “vg call” function of vg toolkit v1.6.1.0 software was used for mutation invocation [59], and a mutation set with high confidence was obtained through quality filtering. For regions with complex variation, the wavelet decomposition method was adopted to analyze multi-allelic and nested variations. Finally, bcftools v1.19 software was used to statistically analyze the variation results [60]. After construction, the mitochondrial pan-genome map was visualized based on SV using Bandage v0.9.0 software [41], and the topological structure of the map was analyzed using ODGI v0.9.2 to evaluate its complexity and connectivity [61].

## 5. Conclusions

In this study, the first graph-based mitochondrial pan-genome of *H. marmoreus* was constructed by integrating 31 mitochondrial genomes (98,284–111,087 bp). The analysis revealed eight gene rearrangement patterns and five genetic clusters, and the resulting graph-based pan-genome was 220,364 bp in length and comprised 217 nodes, thereby systematically uncovering the structural diversity and adaptive evolution of its mitochondrial genome. The identified gene rearrangement patterns, genetic clusters, and abundant genetic variations provide breeding-relevant genetic markers and a genomic framework for *H. marmoreus* germplasm resource improvement, molecular breeding, and molecular regulation. For the cultivation of edible fungi, the results of this study provide a new molecular regulation strategy for improving the cultivation efficiency of *H. marmoreus*; in terms of germplasm preservation, it provides a scientific basis for the classification, identification, and core germplasm selection of *H. marmoreus* germplasm resources. In addition, the graph-based mitochondrial pan-genome construction method used in this study can be extended to other edible and medicinal fungi, providing a new technical approach for pan-genome research on and the molecular breeding of edible fungi.

## Figures and Tables

**Table 1:** Summary of mitochondrial genome assembly characteristics of 31 *Hypsizygus marmoreus* strains.

Number	Size (bp)	Introns	Exons	Total Genes	Protein-Coding Genes	No. of rRNAs	No. of tRNAs	GC Contents %	Accession No.	Source Information
1	104,282	10	14	78	48	2	28	31.52	nn12-1	This study
2	104,284	10	14	74	46	2	28	31.52	nn12-17	
3	106,605	10	14	75	47	2	27	31.72	f2	
4	106,609	10	14	76	47	2	27	31.72	f4	
5	98,284	7	9	65	39	2	28	31.75	SRR7874787	[2]
6	102,752	9	13	72	44	2	27	31.86	MH382825.1	[6]
7	106,518	10	14	77	49	2	27	31.67	MF133443.1	
8	106,608	10	14	75	47	2	27	31.72	SRR8699805	
9	106,718	10	14	75	47	2	27	31.77	SRR8699833	
10	105,706	10	14	74	47	2	25	31.64	SRR8699796	
11	106,610	10	14	75	47	2	27	31.72	SRR8699803	
12	106,773	10	14	75	47	2	27	31.75	SRR8699801	
13	106,699	10	14	76	48	2	27	31.77	SRR8699802	
14	106,677	10	14	75	47	2	27	31.75	SRR8699837	
15	106,604	10	14	75	47	2	27	31.72	SRR8699817	
16	106,606	10	14	75	47	2	27	31.73	SRR8699808	
17	106,608	10	14	75	47	2	27	31.72	SRR8699813	
18	106,711	10	14	76	48	2	27	31.75	SRR8699800	
19	106,612	10	14	76	48	2	27	31.73	SRR8699835	
20	106,699	10	14	75	47	2	27	31.74	SRR8699816	
21	106,691	10	14	75	47	2	27	31.75	SRR8699834	
22	106,609	10	14	75	47	2	27	31.72	SRR8699814	
23	106,620	10	14	75	47	2	27	31.73	SRR8699815	
24	105,589	10	14	74	48	2	25	31.61	SRR8699809	
25	106,609	10	14	69	41	2	27	31.72	SRR8699804	
26	106,603	10	14	75	47	2	27	31.72	SRR8699797	
27	106,602	10	14	69	41	2	27	31.72	SRR8699811	
28	111,087	9	12	74	46	2	27	32.12	SRR12151860	[4]
29	106,670	10	14	75	47	2	27	31.74	SRR12151875	
30	111,031	9	12	75	47	2	27	32.11	SRR12151871	
31	111,037	9	12	74	46	2	27	32.11	SRR12151883	

## Data Availability

The public genomic information of strains f2, f4, nn12-1, and nn12-17 can be found, respectively, in the GenBank database (PV946885, PX600725, PX600726, and PX600727). At the same time, the code related to this article was uploaded to GitHub (GitHub—maruichenzhanghao/Hypsizygus-marmoreus-mito-Pan-genome: A graph-based pan-genome constructed from the mitochondrial genomes of 31 *Hypsizygus marmoreus* strains).

## Supplementary Materials

The following supporting information can be downloaded at https://www.mdpi.com/article/10.3390/ijms27073129/s1.
