# Supplementary material for: Mitochondrial Graph-Based Pan-Genome Analysis of Hypsizygus marmoreus: Structural Variation, Adaptive Evolution, and Its Implications for Germplasm Resource Improvement
Source: Int J Mol Sci. 2026 Mar 30;27(7):3129. doi: 10.3390/ijms27073129 (PMC13073876; doi:10.3390/ijms27073129)
Supplement: Supplementary file 1 [file ijms-27-03129-s001.zip › ijms-4189155-supplementary.pdf]

International Journal of
Molecular Sciences

MDPI

Supplementary Material

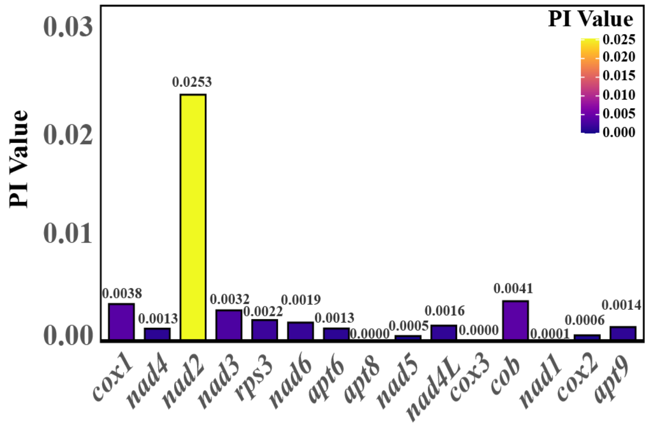

Figure S1. Analysis of nucleic acid diversity in the mitochondrial genome of *Hypsizygus marmoreus*. Analysis of nucleotide polymorphisms at the PCG gene level (window length 100 bp, step size 25 bp).

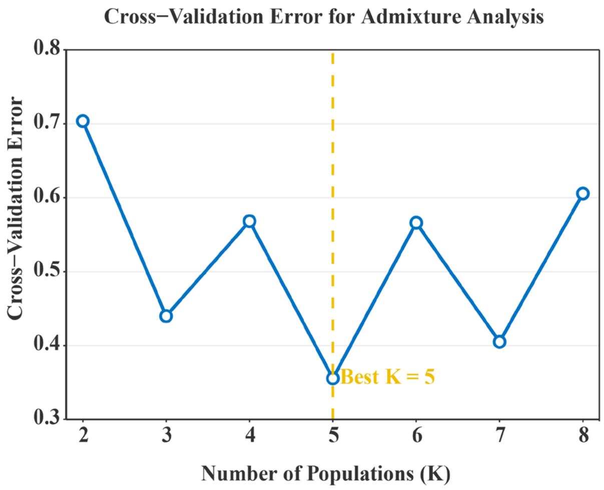

Figure S2. Cross-validation error from ADMIXTURE analysis.

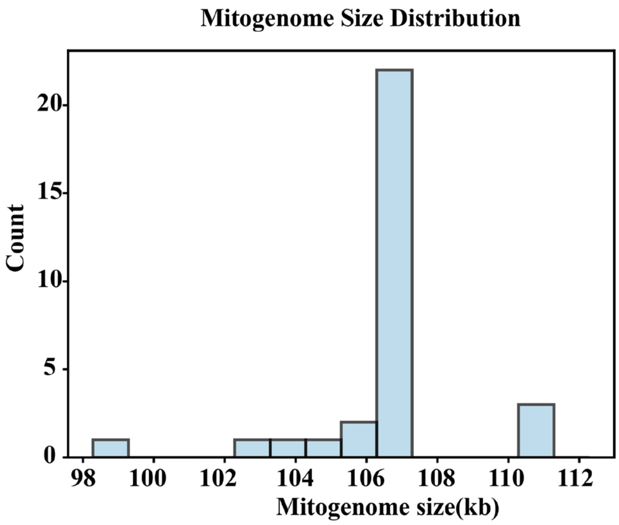

Figure S3. Variation in genome length among the 31 strains.

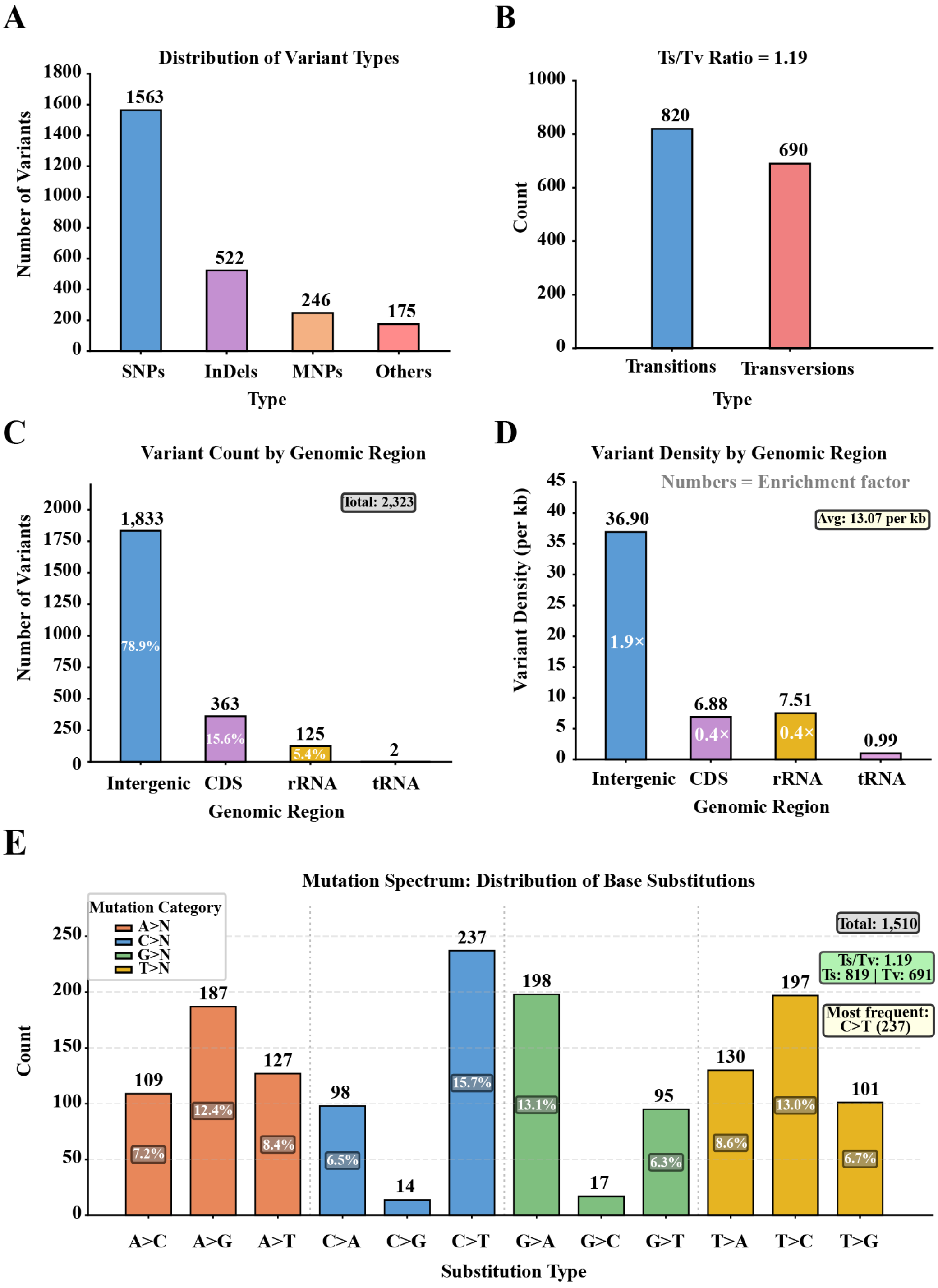

Figure S4. Characterization of genomic variants and mutation spectrum in *Hypsizygus marmoreus* mitogenomes. (A) Distribution of variant types: SNPs, InDels, MNPs, and others; (B) transition (Ts) vs. transversion (Tv) counts; (C) number of variants distributed across genomic regions: intergenic, CDS, rRNA, and tRNA; (D) variant density across genomic regions; (E) mutation spectrum showing distribution of base substitution types.
